# Sensory signals for nausea

**DOI:** 10.1016/j.tins.2025.08.003

**Published:** 2025-09-18

**Authors:** Shiling Hu, Ashley Loureiro, Chuchu Zhang

**Affiliations:** 1 Department of Physiology, David Geffen School of Medicine, University of California, Los Angeles, Los Angeles, CA, USA; 2 These authors contributed equally

## Abstract

Nausea serves as a protective response against harmful ingested stimuli but can also be experienced as a discomforting aspect of various conditions. Recent insights have emerged regarding neural pathways and molecular mechanisms linked to this sensation. This often involves complex interactions of interoceptive neural pathways with the digestive, endocrine, and immune systems. This review summarizes recent findings using non-emetic (e.g., rodents) and emetic (e.g., ferrets, shrews, dogs) mammalian models to explore the molecular mechanisms of nausea, particularly in understudied malaise states. By investigating how nausea is triggered across different contexts, we aim to clarify the general sensory principles governing this response and to promote a shift in therapeutic research – from a top-down, observational paradigm to a bottom-up, mechanism-driven approach.

## Understanding nausea: from gut defense to disease

The ability to avoid harmful substances from entering the digestive tract is universally crucial for survival and homeostasis [1-3]. The first line of defense against potentially harmful substances, such as spoiled food, involves the external senses of smell, taste, and sight. If dangerous substances are ingested, the internal sensory system triggers protective responses, causing nausea and vomiting. Nausea, an uncomfortable feeling of distress associated with the digestive system, often leads to vomiting, or ‘emesis’, to eliminate the ingested material toxins [4]. Nausea promotes avoidance learning and aversive memory in both humans and animals, resulting in avoidance of external cues associated with the nausea experience. Besides external sensory cues and visceral inputs, psychological states such as stress and anxiety can also serve as triggers [5]. Nausea is also one of the most prevalent maladaptive side effects associated with medications, particularly those used for the treatment of cancer and obesity [6,7]. Nausea also occurs during pregnancy, motion sickness, anesthesia, food allergies, gastrointestinal disorders, and migraines [8].

In the pursuit of understanding nausea, researchers have utilized many different experimental animal models [9]. These include species that are capable of emesis, such as ferrets, shrews, and dogs [9,10]. However, the paucity of genetic tools for these emetic species presents a significant obstacle to gaining further mechanistic insights. Commonly used rodent models do not have an emesis reflex. However, human nausea-inducing agents (emetics) administered to rodents cause reactions that include anorexia, sickness behaviors, and a powerful avoidance of paired cues. These reactions can be blocked by anti-emetics, suggesting evolutionarily conserved cellular and neuronal mechanisms [9,11]. These rodent models offer, therefore, important tools to help advance current understanding of the neural and sensory mechanisms of nausea.

Recent studies leveraging advances in mouse genetics tools, molecular techniques, and human genetics show that nausea and vomiting result from complex interactions between interoceptive sensory systems and the digestive, immune, and endocrine systems. This review seeks to summarize current understanding of central and peripheral neural pathways and molecular mechanisms of nausea across various contexts. We also outline directions for further research and venues for the development of anti-emetics grounded in the mechanistic understanding of nausea.

## Neural pathways for nausea

Signals relevant to nausea are first detected through several sensory pathways located in the peripheral nerves and in the brain. Apart from motion-related stimuli, which are mainly sensed through the vestibular pathway [12,13], the majority of nausea signals are likely detected through the area postrema and vagal afferent pathway.

The area postrema is a sensory circumventricular organ located in the brainstem with direct access to peripheral circulation and cerebrospinal fluid [14,15]. Historically known as the chemoreceptor trigger zone for nausea, the area postrema is anatomically privileged to sense nausea-related stimuli in the blood to induce emesis [14,15]. The area postrema comprises both excitatory and inhibitory neuronal types. Single-cell transcriptomic analyses have identified two predominantly distinct excitatory populations characterized by the expression of glucagon-like peptide 1 receptor (GLP1R) and calcitonin receptor (CALCR), respectively [16]. Within the GLP1R^+^ population, at least two smaller excitatory subclusters have been described – one marked by the expression of GFRAL, the receptor for growth differentiation factor 15 (GDF15), and another expressing the norepinephrine transporter, SLC6A2. Stimulation of either these subpopulations (GFRAL^+^ or SLC6A2^+^) or the broader GLP1R^+^ group induces conditioned flavor avoidance behavior in mice [16,17]. Inhibitory neurons in the area postrema are also characterized by the expression of receptors such as the glucose-dependent insulinotropic polypeptide receptor (GIPR) [17]. Specific genetic ablation of the GLP1R^+^ neuronal populations in the area postrema of mice abolishes avoidance conditioned to nausea-related cues, including exendin-4 (GLP1R agonist), bacterial lipopolysaccharide, and lithium chloride (gut irritant) [16,18]. Conditioned GLP-1R knockout in the area postrema abolishes the exendin-4-induced conditioned taste avoidance [19]. Blocking GFRAL receptors in mice using a monoclonal antibody alleviates the adverse side effects of cisplatin [20]. These receptor-specific loss-of-function experiments support a critical role of the area postrema as a primary sensory site for detecting nausea-inducing signals. Anatomically, GLP1R^+^ neurons in the area postrema project to calcitonin gene-related peptide (CGRP)-expressing neurons in the parabrachial nucleus (PBN). By contrast, CALCR^+^ neurons send projections to CGRP-negative regions of the PBN [16].

The vagus nerve is another key sensory pathway for nausea. Stimulating the vagus nerve promotes emesis in anesthetized animals, while cutting the vagus nerve innervating the digestive tract abolishes nausea in response to several ingested toxins but not to motion [21-23]. These findings suggest that the gut-innervating vagal afferents contribute to sensing nausea signals in the viscera. In particular, the vagus nerve serves as a major gut–brain connection in detecting nausea cues in the digestive tract, including irritants and noxious stretches [21,24]. Vagal sensory neurons are molecularly heterogeneous and functionally distinct [25-27]. Single-cell mapping has revealed that vagal afferents comprise diverse subtypes with specialized roles in detecting mechanical and chemical signals in various thoracic and abdominal organs. For example, G protein-coupled receptor 65 (GPR65)-expressing vagal sensory neurons detect intestinal nutrients, while the GLP-1R-expressing population detects stomach stretch [25,28,29]. However, the identities of vagal sensory neurons that mediate nausea remain elusive.

The vagal sensory neurons relay visceral information to both the brainstem caudal nucleus of the solitary tract (cNTS) and the area postrema. The cNTS is a key visceral sensory relay station that receives and processes information from the gastrointestinal, respiratory, and cardiovascular systems and plays a crucial role in autonomic reflex and homeostatic functions. Specific visceral inputs engage distinct cNTS subdomains and cell types [30,31]. Through single-cell sequencing and functional mapping, several cNTS neuronal populations that may be relevant to nausea have been examined. This includes the tachykinin precursor 1 (TAC1)^+^, calbindin 1 (CALB1)^+^ , and cholecystokinin (CCK)^+^ neuronal types [32-34]. Like area postrema GLP1R^+^ neurons, TAC1^+^, CALB1^+^, and CCK^+^ cNTS neurons also send projections to CGRP^+^ neurons in the PBN, which are critical for aversive learning mechanisms [16,32,33,35]. In addition, these neurons project to the premotor and motor regions in the brainstem, including the dorsal motor nucleus of the vagus and the nucleus ambiguous [16,32,33]. These distributed brainstem networks likely contain key nodes that coordinate retching and emesis motor reflexes [9]. In species capable of emesis, these complex reflexes include rhythmic abdominal and diaphragm contractions, coordination in breathing, changes in heart rate, reverse peristalsis, delayed gastric emptying, and increases in gastric pressure [36-38]. Recent studies show a ‘reduced’ retch-like reflex after treating mice with staphylococcal enterotoxins such as SEB, SEC1, SED, SEE, and SHE, or through optogenetic activation of specific cNTS neuron subtypes [32,33]. However, some of the same cNTS neurons mediate airway defensive reflexes [39]. The extent of nausea-related motor reflexes in rodents is still not fully clear. Future work to clarify the neuronal types and behavioral reflexes in species capable of emesis is essential to determine the physiological relevance of brainstem neuronal subtypes in mediating nausea responses.

## Molecular signals for nausea

**Figure 1.**
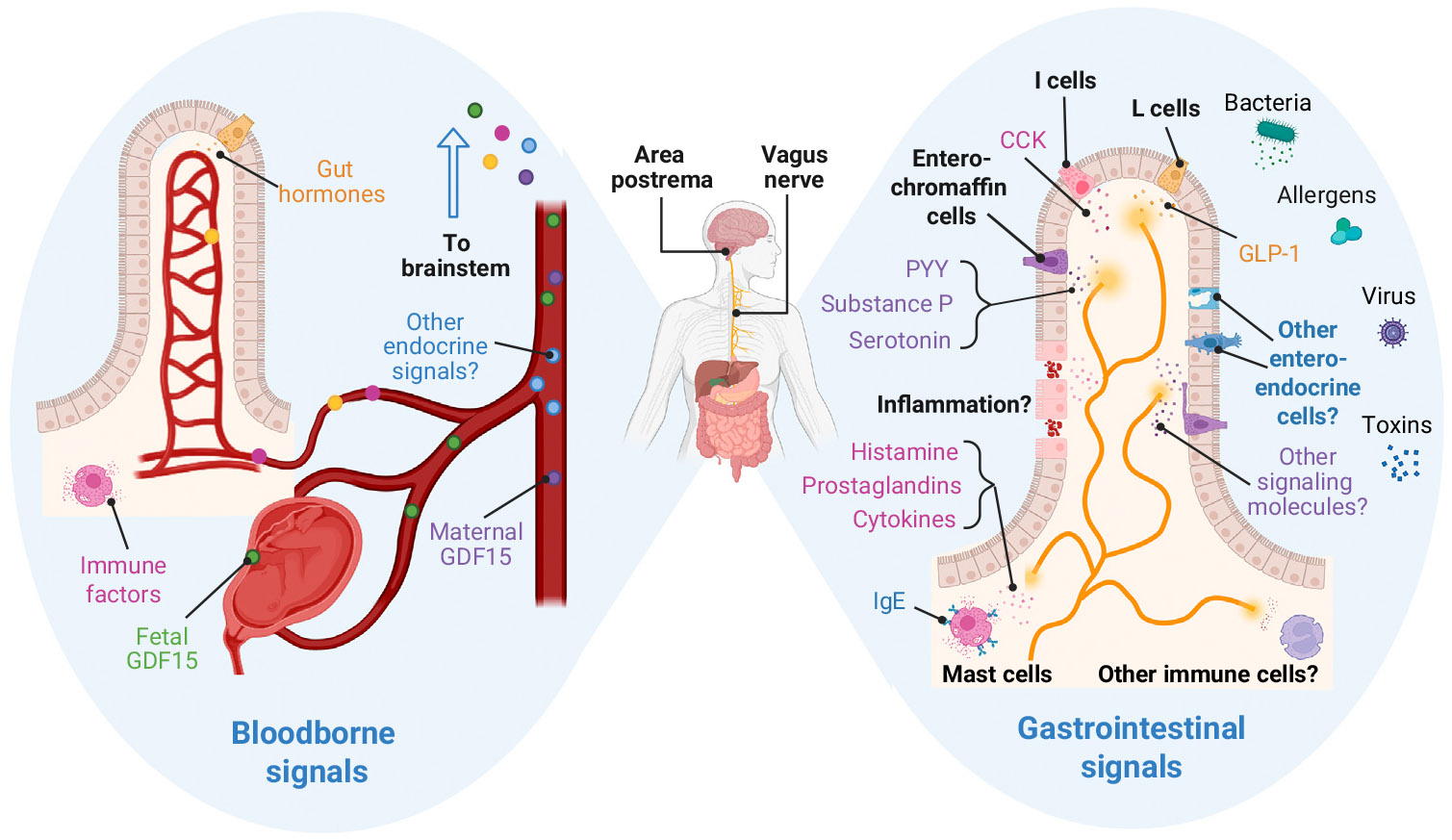
Molecular pathways for nausea. Diverse nausea cues are sensed via both the central nervous system (e.g., area postrema in the brainstem) and peripheral nervous system (e.g., vagus nerve). As a circumventricular region, the area postrema is directly exposed to bloodborne factors. The vagus nerve innervates the gastrointestinal tract and detects ingested factors. Left panel: Known bloodborne signals for nausea include gut hormones, immune mediators (e.g., inflammatory cytokines), and placenta-derived hormones during pregnancy (e.g., GDF15). Right panel: Nausea signals stemming from the ingestion of toxins and microbes activate vagal sensory neurons through various mediators, including enteroendocrine cell-secreted factors (e.g., PYY, substance P, serotonin, CCK, GLP-1), mast cell-secreted factors (e.g., histamine, prostaglandins, cytokines), and potentially inflammatory factors released during gut barrier insult (e.g., cytokines IL-6 and TNF-α, possibly IL-25 and TSLP). Abbreviations: CCK, cholecystokinin; GDF15, growth differentiation factor 15; GLP-1, glucagon-like peptide 1; IL-6, interleukin-6; IL-25, interleukin-25; PYY, peptide tyrosine; TNF-α, tumor necrosis factor alpha; TSLP, thymic stromal lymphopoietin. Figure created using BioRender.com.

Several studies aimed to clarify which nausea-related signals are detected by sensory pathways, and whether these signals are distinct or shared across various conditions. Overall, distinct signaling molecules appear to be involved in specific conditions, yet some are engaged across several conditions. In this section, we summarize these findings (see also Figure 1). We also touch on other possible mechanisms that might be involved.

### Gut hormones

As one of the physiological responses sometimes described colloquially as ‘gut feelings’, nausea has been associated with the influence of gut hormones. Several satiety-promoting gut hormones can trigger nausea when administered pharmacologically in experimental settings, although hormones naturally released in the body may not act in the same way. One of the most notable satiety hormones that causes intense nausea are the analogs of GLP-1. GLP-1R agonists are used to treat obesity and type 2 diabetes, producing significant weight loss and metabolic improvements. However, their clinical application is limited by frequent gastrointestinal side effects, particularly nausea and vomiting, which affect a substantial proportion of patients and impact long-term adherence [40,41]. While GLP-1R agonists act through multiple sites in the peripheral organs and central nervous system, mechanistic studies have revealed that the adverse effects are likely to be mediated exclusively by GLP-1R-expressing neurons in the brainstem, particularly within the area postrema. Notably, in mice, suppression of food intake persists even when the area postrema’s aversive signaling is ablated, suggesting that nausea is not essential for the anorectic efficacy of GLP1R agonists [18]. Distinct GLP-1R circuits, such as those in cNTS and hypothalamus, can suppress appetite through non-aversive, satiety-like mechanisms [18,42-45]. Further research is needed to elucidate whether endogenous sources of GLP-1 signaling, either from the gut or from brainstem glucagon neurons, can be detected by these nausea-promoting area postrema neurons. It is also unclear how long-term therapeutic use of GLP-1 agonists may alter the neuronal properties of the area postrema, particularly in terms of tolerance and sensitivity.

In addition to GLP-1, other gut hormonal signals implicated in nausea include CCK and neuropeptide Y. CCK is a gut-derived peptide hormone from enteroendocrine I cells, released in response to the ingestion of nutrients, particularly fats and proteins. While physiological levels of CCK contribute to normal meal termination, infusing CCK has been shown to induce nausea and emesis in monkeys and humans [46,47]. It is unclear which neural pathway is directly responsible for the aversive effects of CCK, with possible involvement of vagal sensory and motor pathways, neurons in the cNTS, or through the actions of vasopressin hormonal release [46,47]. Vasopressin is another peptide hormone with variable efficacies in triggering nausea and vomiting by regulating stomach muscle tone and sympathetic responses [48-50]. Finally, intestinal neuroendocrine L cells secrete peptide YY, which is hydrolyzed rapidly to PYY (3-36). PYY (3-36) is a potent emetic peptide when administered intravenously in dogs, minks, and humans [51-56]. PYY (3-36) primarily targets the neuropeptide Y_2_ receptor subtype expressed in vagal sensory neurons and the brainstem area postrema, with a possible contribution to nausea from the area postrema [57].

### The alarm signal GDF15 and its role in pregnancy

GDF15 is a cell stress cytokine signal that is elevated during numerous conditions, such as cancer, liver injury, mitochondrial diseases, and exercise [58,59]. Under normal conditions, circulating levels of GDF15 are low but rise sharply in response to cellular stress, inflammation, mitochondrial dysfunction, infection, and cancer, serving as an ‘alarm signal’. The receptor for GDF15 is a complex composed of GFRAL and RET, a receptor tyrosine kinase, which are explicitly expressed in the neurons of area postrema and the adjacent cNTS [58]. Activation of the GDF15-GFRAL pathway robustly suppresses food intake, induces a conditioned taste avoidance, and directly induces vomiting in species capable of emesis, including humans [60,61]. Besides causing nausea and vomiting, this neural pathway also plays a role in systemic glucose and triglyceride metabolism and can induce hypothermia [19,62]. GDF15 signaling plays a significant role in cancer chemotherapy-induced anorexia [58]. It also mediates the effects of metformin on body weight reduction [63].

A series of genetic-associated studies in humans have implicated GDF15 in pregnancy-induced nausea [64-66]. Nausea and vomiting affect approximately 70% of human pregnancies. These responses likely serve as a protective mechanism during early pregnancy to guide food choices to avoid potential teratogens. However, in about 1–2% of pregnancies, nausea and vomiting are so severe that women are unable to eat and/or drink normally, a condition named hyperemesis gravidarum, which is among the leading reasons for hospitalization during pregnancy [67].

Genome-wide association studies of women with hyperemesis gravidarum reported several independent variants proximal to the GDF15 gene as the most highly associated single nucleotide polymorphisms in the maternal genome [64,65]. An exome sequencing study in hyperemesis gravidarum cases found a rare, heterozygous missense variant in GDF15 (C211G) highly enriched in hyperemesis gravidarum cases versus controls, with a tenfold increased risk of developing the condition [66]. This C221G variant is associated with lower circulating levels of GDF15 in the non-pregnant state [68]. During pregnancy, the fetoplacental unit is the major source of GDF15 in maternal blood, circulating fetal-derived GDF15 [68]. Mothers carrying the C221G variant are more sensitive to sharp increases in fetal-derived GDF15 due to long periods of low base-line circulating GDF15 levels, leading to severe nausea. Conversely, for mothers with chronically high levels of GDF15 (e.g., those with beta-thalassemia), the incidence of nausea and vomiting during pregnancy is much reduced [68]. Hence, the severity of nausea and vomiting during pregnancy is the result of the interaction of fetal-derived GDF15 and the mother’s sensitivity to this peptide through its receptor GFRAL in the area postrema established prior to pregnancy.

GDF15/GFRAL desensitization also provides a plausible explanation for the subsiding of nausea after the first trimester [68]. It remains uncertain whether pregnancy hormones, such as human chorionic gonadotropin or estrogen, along with other endocrine factors, contribute to nausea during this early phase of pregnancy. Interestingly, nausea is not constant during early pregnancy. It is significantly influenced by daily activities, particularly feeding. How the neural mechanisms of nausea may be influenced by other sensory modulations during pregnancy remains to be clarified. Future work may also explore therapeutic strategies involving GDF15 signaling that might help relieve nausea during pregnancy.

### Neurotransmitters as nausea signals in food poisoning

Nausea and vomiting are early warning signs of food poisoning. A myriad of nauseating agents contaminate our food, including bacterial exotoxins, viruses, fungi, and plant-defensive alkaloid compounds. Studies showed that cutting the subdiaphragmatic vagus nerve abolishes nausea and vomiting in response to various food poisoning toxins such as copper sulfate and staphylococcal enterotoxin, but not to motion, establishing the importance of the vagal pathway for detecting ingested toxins [22,23,69-71]. These toxins are largely proteinaceous or small molecules in nature. This wide array of nauseogenic molecules likely evokes nausea through distinct mechanisms by directly or indirectly activating the vagal neurons innervating the gut. Some molecules may also engage bloodborne signals that engage the area postrema.

One signaling pathway that has been highly implicated between gut barrier cells and the vagus nerve involves serotonin. In the gut barrier, serotonin is primarily synthesized by enterochromaffin cells, a type of enteroendocrine cells lining the gut, and is released as a neurotransmitter that can directly stimulate vagal sensory terminals. The majority of the vagal sensory neurons express the ionotropic serotonin receptor (5-HT3) genes (HTR3A and HTR3B) [27]. Activating enterochromaffin cells in mice induces avoidance [72]. Antagonists for the 5-HT3 receptor are widely used as anti-emetics, suggesting a possible role of serotonin signaling in inducing nausea, although the exact site of action is still unclear [73]. How gut barrier cells detect harmful substances to release serotonin is a key area of research.

The serotonergic enterochromaffin cells express members of the transient receptor potential (TRP) ion channel family, such as transient receptor potential ankyrin 1 (TRPA1) [74], that detect reactive electrophilic irritants [75]. These TRPA1^+^ enterochromaffin cells are primarily located in the crypts of the intestine. Strong electrophiles, like acrolein, penetrate the mucosal barriers and stimulate TRPA1 to promote serotonin release [76]. Some of the cells located in the villi of the intestine also express transient receptor potential melastatin 2 (TRPM2), which senses oxidative stress chemicals such as reactive oxygen species through the detection of intracellular ADP ribose [76]. Notable examples include hydrogen peroxide, a commonly used emetic for veterinary practices [77]. Moreover, cancer chemotherapy drugs may generate reactive oxygen species or acrolein [76-78]. This may extend to irritable bowel syndrome-related conditions where reactive chemicals are also generated to engage the enterochromaffin-vagal pathway via serotonin signaling to induce nausea, in addition to other digestive symptoms [77].

During viral gastroenteritis, also known as ‘stomach flu’, an inflammatory gut infection caused by highly contagious viruses results in nausea and acute vomitin g [79]. Rotavirus expresses the viroporin NSP4, an ion channel protein that forms a hydrophilic pore in cell membranes to modify calcium ion permeability and homeostasis [79]. Following rotavirus infection, enterochromaffin cells exhibit increased cytosolic calcium ions and, in response, release serotonin [80,81]. Infection of rotavirus activates immediate early gene cFos in the cNTS [81]. In addition to rotavirus, norovirus has been found to infect intestinal enteroendocrine cells of human patients [82]. Whether norovirus infection also results in increased serotonin release by these infected cells and activation of the dorsal vagal complex remains to be determined.

Bacterial exotoxins are also common nausea-inducing signals. These include, for example, the foodborne pathogen *Bacillus cereus*, which secretes cereulide, and *Staphylococcus aureus*, which secretes staphylococcal enterotoxins [83,84]. In mice, blocking serotonin receptors has been shown to reduce emesis in response to staphylococcal enterotoxins and cereulide [23,33,85]. The mechanisms of serotonin induction, however, require further investigation; these potentially include injury-related signaling [33]. Exposure to cereulide in mice was shown to stimulate immediate early gene cFos in downstream cNTS neurons expressing CALB1, while staphylococcal enterotoxins stimulate cFos in those expressing TAC1 [32,33]. It is unclear how vagal sensory neurons responding to serotonin ultimately stimulate these two distinct populations of cNTS neurons. Notably, there are regional differences in enterochromaffin populations along the digestive tract [86]. Whether these are involved in the differential responses in the cNTS remains to be examined.

A generalized signaling pathway involving only serotonin is unlikely to account for all forms of nausea-related toxin sensing. For example, histamine may act as a key neurotransmitter during food poisoning. Scromboid food poisoning is caused by the ingestion of seafood containing high exogenous levels of histamine that is accumulated from converted histidine during food spoilage [87]. Similarly, with staphylococcal enterotoxin ingestion, mast cells also release histamine, which, along with serotonin, is a neurotransmitter relevant for emesis in musk shrews [85]. However, it is unclear which neural pathway is responsible for histamine-induced nausea, with histamine receptors expressed in multiple sites, including the dorsal vagal complex, intestinal enteric neurons, and intestinal epithelial cells [88,89].

Poisoning by ingestion of several type A trichothecene mycotoxins results in elevations of substance P in the plasma, which is secreted by enterochromaffin cells of the gut [90]. Substance P can bind to neurokinin-1 receptors expressed peripherally in somatosensory neurons innervating the gut and centrally in neurons of the cNTS and dorsal motor nucleus, where its emetic action is likely through central stimulation [91,92]. Peripherally, mycotoxins may also enter the submucosal space via paracellular and transcellular routes, where they can directly interact with sensory neurons in the gut [93,94]. Some toxins may also trigger injury responses, where proinflammatory cytokines mediate the nausea responses. For example, cytokines are also elevated during food poisoning by mycotoxin deoxynivalenol (IL-6, IL-1β, TNF-α, and IL-18) and staphylococcal enterotoxin (IL-1β and IL-18) [95,96]. Cytokines such as IL-6 and TNF-α may cause nausea by interacting with vagal and area postrema neurons, as discussed later regarding inflammation contexts (see ‘Immune activation’). Future research in these areas may provide important insights into interoceptive noxious sensing via as yet undetermined signaling mechanisms.

### Immune activation

Nausea is traditionally viewed as a protective response triggered by mechanical or chemical signals such as gastrointestinal distension or toxins. However, growing evidence suggests that immune activation can also elicit nausea-like responses. Gastrointestinal symptoms, including nausea, vomiting, and diarrhea, are common among patients with food allergies, digestive diseases, cancer, and other conditions [97,98]. Immune activation can drive nausea through multiple mechanisms, with a common feature being the interaction between inflammatory mediators and sensory neurons.

Mast cells are important downstream effectors leading to neuronal responses during food allergies, especially for the IgE-mediated food allergy type. Re-exposure to antigens causes the rapid release of mediators, such as histamine, prostaglandins, serotonin, and leukotrienes [99,100]. Studies in mice have shown that in the gut, mast cells are located close to neurons in the intestinal mucosa, with increased colocalization during food allergy [101,102]. The activation of mast cells is critical for the behavioral avoidance of allergens and visceral pain responses [103-105]. In a mouse model of ovalbumin allergy, sensitization of the constituent protein ovalbumin results in avoidance of ovalbumin-spiked drinking water [103,104]. This behavior relies on the involvement of IgE, mast cells, and cysteinyl leukotrienes, which stimulate brain regions such as the cNTS, PBN, and amygdala [103,104]. It is unclear whether the mast cell-to-neuron signaling in the gut engages both pain and nausea pathways in the food allergy models, or whether nausea may be mediated via other mechanisms. For example, GDF15 also increases during the effector phase of food allergy, which can cause aversion [104]. In food allergy patients, besides IgE-mediated, non-IgE-mediated allergies involve distinct mechanisms, with some causing severe nausea symptoms [97]. For example, non-IgE-mediated allergies can affect intestinal epithelial barrier disruption [106,107], local inflammation, and the release of distinct cytokines (such as IL-25, TSLP). Whether these effector cells and factors act through vagal afferents or the area postrema is unclear.

Systemic infection and sepsis are often accompanied by profound gastrointestinal symptoms such as malaise, nausea, and vomiting [108,109]. Over 20 years ago, pioneering mouse studies demonstrated that broad electrical stimulation of the whole vagal nerve bundle can protect animals from shock produced by high levels of TNFα [110,111]. In mice, vagal sensory neurons directly respond to cytokines, and activating TRPA1-expressing vagal neurons dramatically enhances the anti-inflammatory response and severely suppresses the levels of proinflammatory cytokines. TRPA1^+^ vagal neurons send inputs to the cNTS and stimulating these cNTS neurons also suppresses LPS-induced IL-6, IL-10, IL-1β, and TNFα [112,113]. These studies demonstrate that infection-related cytokines can stimulate vagal sensory circuits, which in turn modulate immune responses. Whether these neurons also trigger nausea-related responses has yet to be clarified.

Cancer cachexia is a devastating metabolic wasting syndrome characterized by anorexia, fatigue, and dramatic involuntary bodyweight loss [114,115]. It induces a chronic elevation of IL-6, which can rapidly enter the area postrema and activate associated downstream networks to promote anorexia, catabolic metabolism, and apathy (low motivation state) [116,117]. Neutralization of IL-6 in the brains of tumor-bearing mice, via intracerebroventricular infusion of an IL-6 antibody, attenuates cachexia and the cachexia-associated hyperactivity within the area postrema network [116].

Collectively, these findings highlight the close interplay between immune cell activation, mediator release, and interoceptive circuits as seen in food allergy, inflammation, and cancer cachexia, which are clinically significant triggers of nausea and malaise. Future studies dissecting these pathways will be critical for uncovering how immune signals drive nausea and for guiding mechanism-based therapeutic strategies.

## Anti-emetics: from reverse translational to a precision approach

The design of contemporary anti-emetics has generally followed a reverse-translational path: existing drugs were found to be concomitantly anti-emetic and were then repurposed for nausea treatment [118]. The efficacy of these compounds in treating nausea also prompted investigation into their mechanisms of action. The earliest anti-emetics discovered are muscarinic antagonists, which are mainly applicable to motion sickness, and act as anticholinergics targeting the vestibular nuclei [119]. Antihistamines and dopamine antagonists are currently the most commonly used first-line treatments for nausea [120-126]. Among them, a common dopamine receptor antagonist, metoclopramide, was found to be more effective due to its action as a selective serotonin 5HT3 receptor antagonist. This has led to the discovery of serotonin 5HT3 receptors as a target for anti-emetics [121,122,125]. Further refinement of selectivity for the 5HT3 receptor resulted in the development of granisetron and ondansetron, both widely used for nausea [127-130]. A small class of analgesics that target substance P receptor (e.g., aprepitant), have been found to possess anti-emetic activity and are thus also narrowly applied as prophylactics for chemotherapy and cyclic vomiting syndrome [127,131]. While not inherently anti-emetic on their own, both corticosteroids (e.g., dexamethasone) and antiepileptics (e.g., topiramate) have been prescribed as combination therapies with the aforementioned anti-emetics [119,124,127,128,131-135]. Currently approved and widely used anti-emetics often provide only limited relief from nausea, particularly in complex clinical conditions such as obesity management, pregnancy, and food allergy, suggesting nausea pathways may be highly context-specific [120,136]. With advancing understanding of the neural mechanisms of nausea, targets such as neuropeptide Y receptor antagonism, anti-GDF15, and GIP agonism may offer new therapies. There is a pressing need to complement the current top-down, observational paradigm with a bottom-up, mechanism-driven approach rooted in neuroscience for developing more effective context-specific anti-emetics.

## Concluding remarks and future perspectives

The area postrema and vagal sensory neurons play crucial roles in detecting nausea cues in visceral systems. They serve as a gateway to midbrain and forebrain regions for sensory processing, which ultimately leads to the sensation of nausea. Nausea can be elicited by various triggers, including environmental factors such as chemicals and physiological stimuli. The internal sensory systems that drive nausea involve complex interactions with other systems, including the digestive, immune, and endocrine systems. Some signaling mechanisms may be utilized across conditions, for example, serotonin signaling between enterochromaffin cells and vagal sensory neurons, and endocrine signaling by GDF15 to the area postrema. Proinflammatory signals may also function as a common mediator, but the details have yet to be clarified. Other signaling mechanisms may be specific to chemical triggers or clinical contexts. Understanding how these peripheral sensory systems detect nausea cues in both homeostatic and disease contexts is a major goal for future research (see Outstanding questions).

Innovations in research tools for studying interoceptive sensory systems allow tackling some of these future questions. These advancements include state-of-the-art *in vivo* and *ex vivo* imaging, assessing organ physiology in combination with targeted manipulations such as optogenetics and chemogenetics, and advanced single-cell sequencing. The rise of high-throughput behavioral phenotyping [137] alongside improved viral tools brings new opportunities for nausea research.

Nausea can seriously impact both the physical and mental well-being of individuals and, in severe cases, it may even lead to significant life changes, such as discontinuation of certain disease treatments or termination of pregnancy. In addition, many existing anti-emetic medications are either ineffective or associated with adverse effects, posing significant risks to vulnerable populations, including pregnant women and the elderly [138]. Further research into nausea will enhance understanding of its molecular mechanisms and lead to better clinical treatments.

## Highlights

Nausea is a protective interoceptive response that integrates external sensory cues, visceral signals, and psychological states across diverse clinical contexts.

Most nausea signals are likely detected through the vagus nerve and the area postrema – which convey signals from the gut and blood circulation to central nausea circuits.

Different physiological and pathological signals activate the vagus nerve and the area postrema through distinct molecular triggers, such as gut-derived hormones, neurotransmitters, and fetal-derived factors.

Immune signals such as inflammatory cytokines are increasingly recognized as upstream contributors to nausea in allergic, inflammatory, and cancer cachexia conditions.

## Outstanding questions

There are distinct types of vagal neurons, molecularly and functionally. Which vagal sensory neurons detect nausea-related signals? How many different nausea-sensing types are there? Are they solely dedicated to sensing nausea stimuli, or do they detect signals across modalities?

What is the relationship between the types of brainstem cells that receive nausea inputs, including various cNTS neurons, and neurons in the area postrema? Additionally, how do these neurons relate to other cNTS cell types that respond to visceral cues, such as food and nutrients? How are these neurons connected locally and with neurons in areas beyond the brainstem?

Are there other endocrine factors beyond GDF15 that contribute to the sensation of nausea during pregnancy?

What mechanisms drive the neuron–immune interactions that cause nausea during food allergy and other inflammatory conditions?

What are the signaling mechanisms in other nausea contexts, such as anesthesia, migraine, and digestive diseases?
